# The Voltammetric Detection of Cadaverine Using a Diamine Oxidase and Multi-Walled Carbon Nanotube Functionalised Electrochemical Biosensor

**DOI:** 10.3390/nano13010036

**Published:** 2022-12-22

**Authors:** Mohsin Amin, Badr M. Abdullah, Stephen R. Wylie, Samuel J. Rowley-Neale, Craig E. Banks, Kathryn A. Whitehead

**Affiliations:** 1 Faculty of Engineering and Technology, Liverpool John Moores University, Liverpool L3 3AF, UK; 2 Faculty of Science and Engineering, Manchester Metropolitan University, Manchester M1 5GD, UK; 3 Microbiology at Interfaces Group, Manchester Metropolitan University, Manchester M15 6BH, UK

**Keywords:** biosensor, cadaverine, electrochemistry, multi-walled carbon nanotubes, periodontitis

## Abstract

Cadaverine is a biomolecule of major healthcare importance in periodontal disease; however, current detection methods remain inefficient. The development of an enzyme biosensor for the detection of cadaverine may provide a cheap, rapid, point-of-care alternative to traditional measurement techniques. This work developed a screen-printed biosensor (SPE) with a diamine oxidase (DAO) and multi-walled carbon nanotube (MWCNT) functionalised electrode which enabled the detection of cadaverine via cyclic voltammetry and differential pulse voltammetry. The MWCNTs were functionalised with DAO using carbodiimide crosslinking with 1-ethyl-3-(3-dimethylaminopropyl)carbodiimide hydrochloride (EDC) and *N*-Hydroxysuccinimide (NHS), followed by direct covalent conjugation of the enzyme to amide bonds. Cyclic voltammetry results demonstrated a pair of distinct redox peaks for cadaverine with the C-MWCNT/DAO/EDC-NHS/GA SPE and no redox peaks using unmodified SPEs. Differential pulse voltammetry (DPV) was used to isolate the cadaverine oxidation peak and a linear concentration dependence was identified in the range of 3–150 µg/mL. The limit of detection of cadaverine using the C-MWCNT/DAO/EDC-NHS/GA SPE was 0.8 μg/mL, and the biosensor was also found to be effective when tested in artificial saliva which was used as a proof-of-concept model to increase the Technology Readiness Level (TRL) of this device. Thus, the development of a MWCNT based enzymatic biosensor for the voltammetric detection of cadaverine which was also active in the presence of artificial saliva was presented in this study.

## 1. Introduction

Cadaverine, a metabolite found in both eukaryotic and prokaryotic cells, has been demonstrated to have detrimental effects in diseases such as periodontitis [1,2]. Cadaverine belongs to a class of metabolites known as the polyamines [3]. Such molecules have been implicated in inflammatory diseases due to their ability to disrupt host cell-signalling pathways, allowing for the continuation of inflammation without host immune cell intervention [4]. Of particular interest during electrochemical analysis is the oxidation peak of cadaverine, which develops due to the formation of 5-aminobutanal, occurring when cadaverine becomes oxidised by the enzyme diamine oxidase (DAO) [5]. Cadaverine and its oxidation by-products have been described previously in diseases such as periodontitis, causing damage and pain as well as increasing the healthcare costs of the afflicted. The use of substrate specific enzymes to improve the detection of cadaverine through electrochemical analysis is an appealing concept [6]. DAO is an enzyme responsible for the breakdown and oxidation of primary amines, such as cadaverine [7]. Utilising such enzymes as recognition elements for biosensors attracts significant attention due to their high substrate specificity, sensitivity, and ease of bio-conjugation. It is important to be able to detect metabolites such as cadaverine within biofluids, to a high degree of accuracy, whilst maintaining low operation and production costs and short turnaround times for results.

Liquid chromatography mass spectrometry (LC-MS) and high-performance liquid chromatography (HPLC) are techniques that are well-established for the detection and determination of biomarkers such as cadaverine in human biofluids, as well as in foodstuffs, since the detection of such molecules are used as indicators of food spoilage [8,9]. Although these analytical methods are highly accurate and specific, they have significant disadvantages for clinical and industrial applications including high costs and inefficient real-time measurements. HPLC measurements for cadaverine detection, for example, are carried out via fluoresce detection, utilising o-phthaldialdehyde as a derivatising agent [10]. The detection of cadaverine using this complex procedure requires expensive equipment and staff training. Therefore, it is important to develop simpler analytical techniques, for efficient point-of-care measurements in clinical environments.

One method that is gaining significant attention is electrochemical sensing, with particular focus towards biosensors. This discipline allows for the rapid identification of biomolecules within biofluids without the requirement for pre-sample processing. Biosensors are defined based on their method of transduction [11]. Transducers convey a wide range of chemical, physical, or biological reactions into an electrical, measurable signal [12]. Electrochemical biosensors have been studied since the early 1960s and can be either impedimetric, potentiometric, or amperometric [13]. Electrochemical biosensors, where the current is monitored when a fixed potential is applied between two electrodes, has been widely used within the last decade, and such systems have since been miniaturised, to operate with smaller sample volumes of complex matrices [14].

The most commonly used materials for the development of screen-printed electrodes (SPEs) are carbon-based, and the structural and physicochemical properties of such materials have been extensively studied in terms of their electrochemical performance [15,16,17]. Moreover, carbon-based nanomaterials are also being utilised in bio-electrochemical sensing [18]. Compounds such as graphene and other carbon allotropes are one such group of nanomaterials which allow for an increase in electrode surface area, since this has been reported to increase the efficacy of electron transfer, resulting in increased biomolecule loading, selectivity, and sensitivity [19].

Carbon nanomaterials possess features that are particularly effective for use in biosensing platforms [20]. In particular, multi-walled carbon nanotubes (MWCNT) have demonstrated their advantages for being incorporated into sensing devices [21,22,23]. MWCNTs are pseudo-one-dimensional allotropes of carbon, presenting as hollow cylindrical structures with one or more walls (single- or multi-wall, respectively), of nanoscale size. They have major advantages for biosensing applications, such as increased surface area, enhanced electron transfer rates between active centres of enzymes and the electrode, increased stability, and multiple active sites, which enable a high loading capacity for enzymes [24]. One of the major advantages for electrochemical biosensing platforms is that MWCNTs can be used as platforms for the immobilisation of biomolecules [25].

Biomolecule immobilisation is a critical step in ensuring efficient electron transfer between the enzyme active site and the electrode, thus MWCNTs have been utilised for adsorbing enzymes onto their surface structures to create an electron bridge between the working electrode surface and the flavin adenine dinucleotide (FAD) group which is deeply embedded within the centre of the enzymes [26]. Enzyme loading and entrapment, which is an important step in effective bioelectrode design, has been extensively studied [27,28]. Physical adsorption is the simplest and most cost-effective method of manufacturing enzymatic biosensors. Positively charged amino acid residues of enzymes are able to be electrostatically adsorbed onto negatively charged colloidal surfaces on electrodes through simple incubation steps [29]. Although this method is fast and simple, it results in unfavourable enzyme orientations which may negatively impact electron transfer rates. To effectively facilitate enzyme immobilisation onto an electrode surface, a number of pre-functionalisation steps can be implemented to aid in increased enzyme loading. Covalent attachment of proteins onto carbon structures such as MWCNTs is one such method that demonstrates increased sensitivity and selectivity in the development of biological sensing platforms [30].

Electrochemical biosensing technologies which emphasise the detection of cadaverine have been studied within recent years [31,32]. Cadaverine has long been studied for its implications in the quality of foodstuffs, namely fish samples, since the presence of cadaverine is indicative of spoilage. Cadaverine is a product of bacterial decarboxylation of lysine; thus, its identification is typically attributed to putrefaction of tissues and is a reliable indicator of bacterially mediated food spoilage. Whilst cadaverine has been documented in the literature as having strong associations with diseases, in particular those of bacterial origin, there is little current literature on cadaverine biosensors with particular interest towards disease detection and diagnostics. Cadaverine has been studied extensively for its implications in diseases such as periodontitis and traditional techniques [33]. Electroanalytical analysis using biosensors offers a potential new alternative to traditional methods, enabling real-time measures of the active patient disease state using non-invasive means for sample measurement in a clinical setting. Thus, the biosensor tested within this work demonstrates the establishment of an electrochemical biosensor for the detection of cadaverine for applications in healthcare and infection diagnostics.

The aim of this research was to modify and analyse screen-printed electrodes to enable the real time measurement of cadaverine. Such a biosensor could be used in future work to produce a sensitive, low-cost, and highly reproducible sensing platform for applications such as a non-invasive point of care biosensing device for periodontitis diagnostics.

## 2. Experimental

All chemicals used were analytical grade and were used as received from Merck (formerly Sigma-Aldrich), Gillingham, UK without any further purification unless stated otherwise. Electrochemical solutions and cadaverine suspensions were prepared using Type 1 ultra-pure deionised water (Milli-Q, Merck, Gillingham, UK) with a resistivity of 18.2 MΩ cm or greater and were degassed with oxygen-free nitrogen prior to any electrochemical measurements.

### 2.1. Fabrication of Screen-Printed Electrodes

The SPEs were fabricated in-house with the appropriate stencil designs to achieve a 3.1 mm diameter carbon working electrode using carbon graphitic ink (Gwent Electronic Materials Ltd., Pontypool, UK). The graphitic ink was printed using a DEK 248 screen printer machine (DEK, Redcar, UK) onto a polyester flexible film (Autostat, Gloucestershire, UK) with a graphitic ink counter electrode. This layer was cured in a fan oven at 60 °C for 30 min. A dielectric paste (Gwent Electronic Materials, Pontypool, UK) was printed onto the polyester flexible film to cover the connections. After a second curing process at 60 °C for 30 min, an Ag/AgCl reference electrode was printed onto the SPE and cured at 60 °C for 30 min so thatthe SPEs were ready to use [34].

### 2.2. Carboxylation of MWCNTs

An acidic solution containing 7.5 mL H_2_SO_4_ (98%) and 2.5 mL HNO_3_ (70%) was used to introduce carboxyl groups onto the multi-walled carbon nanotube (MWCNT) surfaces. For complete carboxylation, two milligrams of MWCNTs (powdered format) (tube diameter × length:110–170 nm × 5–9 µm, Merck, Gillingham, UK) were placed in an ultrasonic bath in a 2 mL acidic solution for 6 h at 80 °C. The MWCNTs were washed twice with 25 mL of Type 1 water to remove any acid residues and placed in an oven at 60 °C to dry overnight.

### 2.3. EDC-NHS C-MWCNT Coupling

The enzyme diamine oxidase was coupled to the carboxylated multi-walled carbon nanotubes (C-MWCNT) using EDC-NHS. The C-MWCNTs (2 mg) were initially suspended in MES buffer solution (2 mL) and sonicated until homogeneous. The C-MWCNTs were then suspended in 1.2 mL of a 10mg mL^−1^ 1-ethyl-3-(3-dimethylaminopropyl)carbodiimide hydrochloride (EDC) solution (pH 6.5) and left to stand at room temperature for 1 h. *N*-Hydroxysuccinimide (NHS) solution (2.2 mL of a 50 mg mL^−1^) was then added to the C-MWCNT/EDCs and incubated at 37 °C for 1 h under stirring conditions. This coupled the NHS to the pre-conjugated EDC. The coupled C-MWCNT/EDC-NHS were rinsed using 5 mL of 50 mM MES buffer solution (pH 6.5) through a PTFE membrane filter (0.45 µm) to remove any unconjugated residues. The fully conjugated C-MWCNT/EDC-NHS were then dried in a class II cabinet for 1 h before being stored at 4 °C until required.

### 2.4. DAO Conjugated MWCNT Preparation

DAO solution was made using 10 mg/mL DAO in 0.1 M phosphate buffer, and to this, MWCNT/EDC-NHS (2 mg) in 2 mL of MES, (50 mM at pH 6.5) were added and incubated at 37 °C for 1 h under constant shaking (200 rpm) to allow for the enzyme to conjugate with the MWCNT/EDC-NHS. Crosslinking was carried out using 1 mL of a 0.2% glutaraldehyde solution (GA) (Agar Scientific, Stansted, UK), which was added to the C-MWCNT/DAO/EDC-NHS and incubated at room temperature under constant shaking for 30 min, followed by further incubation overnight at 4 °C. The C-MWCNT/DAO/EDC-NHS/GAs were re-suspended in Tris buffer (100 mM at pH 7.2) for 30 min and then washed to remove any unconjugated residues. The C-MWCNT/DAO/EDC-NHS/GA were re-suspended in 0.1 M MES and stored at 4 °C until ready to use.

### 2.5. C-MWCNT/DAO/EDC-NHS/GA Electrode Functionalisation

The working electrode was prepared for functionalisation using the C-MWCNT/DAO/EDC-NHS/GAs through two sequential rinse steps consisting of 2 mL of distilled H_2_O (DH_2_O). Three replicate SPEs were adhered to a Petri dish using 10 mm × 10 mm double-sided tape under sterile conditions to eliminate any potential airborne contaminants. To evenly distribute the C-MWCNT/DAO/EDC-NHS/GA solution onto the working electrode, the C-MWCNT/DAO/EDC-NHS/GA solution was first sonicated for 10 min to homogenise the suspension, and 10 µL was deposited onto the electrode surface using drop-casting, ensuring that the SPEs remained completely unagitated. The modified C-MWCNT/DAO/EDC-NHS/GA SPEs were dried in the class II cabinet for 1 h and placed in individual 5 mL sterile plastic bijous with 1 mL MES buffer solution until ready for use.

### 2.6. Fourier Transform Infra-Red Spectroscopy

Unmodified and modified MWCNTs were analysed using FTIR (PerkinElmer, Buckinghamshire, UK) for new bond examination. MWCNTs at a volume of 15 µL were deposited onto 10 mm × 10 mm silicon wafers (Montco Silicon Technologies Inc., Spring City, PA, USA) and dried in a class II cabinet for 1 h. The samples were stored in a desiccator with silica gel, until ready for use. The FTIR attachment used was a type A MCT detector. The aperture was used at 200 mm × 200 mm, and the spectra of the unmodified and modified electrodes were acquired using Omnic 5.2 software (Thermo Fisher, Winsford, UK).

### 2.7. Energy Dispersive X-ray Spectroscopy (EDX)

To determine the chemical composition of the SPE before and after modification with the MWCNT formulation, EDX (Zeiss, Cambridge, UK) analysis was performed using a EDX Sapphire Si (Li) detector and was quantified using a standardless ZAF algorithm. The atomic weight percentage was used (n = 3).

### 2.8. Electrochemical Measurements

Electrochemical assessment of the C-MWCNT/DAO/EDC-NHS/GA electrodes was performed using an EmStat3 (Palmsens, GA Houten, The Netherlands) computer-controlled potentiostat, using PStrace 5.8 software (Palmsens, GA Houten, The Netherlands). A three-pin SPE connector (Palmsens, GA Houten, The Netherlands) was used as a connector between the electrode and the potentiostat. Measurements were taken using a typical three-electrode system, with a nickel wire counter electrode and an Ag/AgCl reference electrode with the SPEs completing the circuit. Cyclic voltammetric analysis was carried out at a potential window of −0.5 to +1.0 V s^−1^ vs. Ag/AgCl reference with scan rates corresponding to 5 mV s^−1^, 10 mV s^−1^, 15 mV s^−1^, 25 mV s^−1^, 50 mV s^−1^, 75 mV s^−1^, 100 mV s^−1^, 150 mV s^−1^, 250 mV s^−1^, and 500 mV s^−1^. A potential range of −0.3 to +0.7 V s^−1^ was utilised for DPV measurements. All blank measurements were made at room temperature with a supporting electrolyte solution of 0.1 M potassium chloride (KCl) solution and a slightly acidic Britton–Robinson buffer made up of equal parts 0.1 M acetic acid, 0.1 M boric acid, and 0.1 M phosphoric acid.

### 2.9. Statistical Analysis

Statistical analysis of the results was carried out using GraphPad Prism 9 (Stable release, San Diego, CA, USA) and unpaired *t*-tests and one way/two-way ANOVA comparison tests were used. In each instance a *p* < 0.05 was deemed statistically significant.

## 3. Results and Discussion

This study utilised the electrochemical detection of cadaverine as a potential marker for the indication of periodontal disease. Previous studies have demonstrated the effects of increased levels of cadaverine vs. poor patient oral health and deemed cadaverine as an important predictive periodontal biomarker [35]. The current measurement techniques for such biomolecules utilise chromatography which is an expensive and time-consuming practice, requiring sample derivatisation prior to the measurements being obtained. Within the clinical dental environment, periodontal disease assessment remains a practice whereby the traditional techniques of clinical attachment level, bleeding on probing, and pocket depth measurements encompass the gold standard of periodontal disease diagnostics [36]. However, these traditional techniques are qualitative and are dependent on the practitioner’s individual assessment of the disease, and this means that clinicians can only identify the history of the disease. Moreover, this method fails to provide information on current disease activity, the patient’s current oral health, or the risk of potential future periodontal breakdown [33]. Since it is qualitative, the results are also not comparable between dental surgeries. Thus, the requirement for periodontal disease analysis and monitoring is of the utmost importance. The use of biosensors that detect cadaverine and can be used in point-of-care devices would enable the current state of oral health of a patient to be established quantitatively without bias or the need for clinical invasive analysis or expensive chromatography-based analysis.

This work modified the electrode of a carbon screen-printed biosensor using MWCNTs with covalently crosslinked DAO. An essential requirement for any enzymatic biosensing device is the immobilisation of the protein which in this instance was achieved through covalent binding to reduce the enzymatic response time and increase the sensor shelf life [37]. In order to carry this out, 1-ethyl-3(3-dimethylaminopropyl)carbodiimide (EDC), a water-soluble zero-length crosslinker, was utilised in the development of the sensing platform [38]. It is typically used in the coupling of carboxyl groups, which in this study were acid-etched onto the MWCNT surface and used to conjugate EDC to primary amines as previously shown [39]. EDC undergoes nucleophilic substitution in the presence of strong nucleotides, such as primary amine molecules, and forms an unstable O-acylisourea intermediate [40]. This intermediate is readily hydrolysable, and thus able to rapidly revert to its original carboxylate molecule. To overcome this, *N*-hydroxysuccinimide (NHS) has been frequently used to develop a more constant intermediate prior to amine introduction [41,42]. This method provides a stable electrode with a greater effective area enabling increased protein loading for maximizing the probability of enzyme–substrate complex formation and overall increased sensitivity [43].

**Figure 1:**
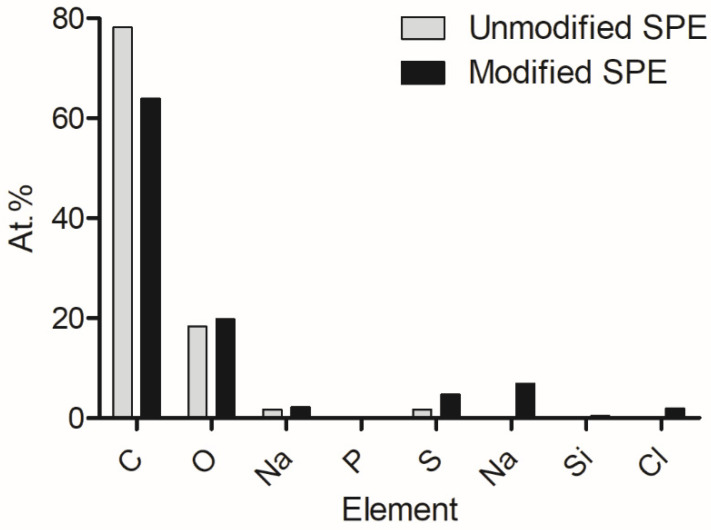
Changes in elemental composition which occurred on the electrode as a result of electrode modification [40].

Following from previous work, energy-dispersive X-ray analysis was conducted to show the elemental composition of the pre- and post-modified electrode surfaces (Figure 1). It was determined that carbon was the bulk element present on the surfaces, and oxygen, sodium, phosphorus, and sulphur were included. However, after modification, nitrogen, silicon, and chlorine were present. These additional elements were suggested to have been introduced via the enzyme, EDC, and NHS compounds.

**Figure 2:**
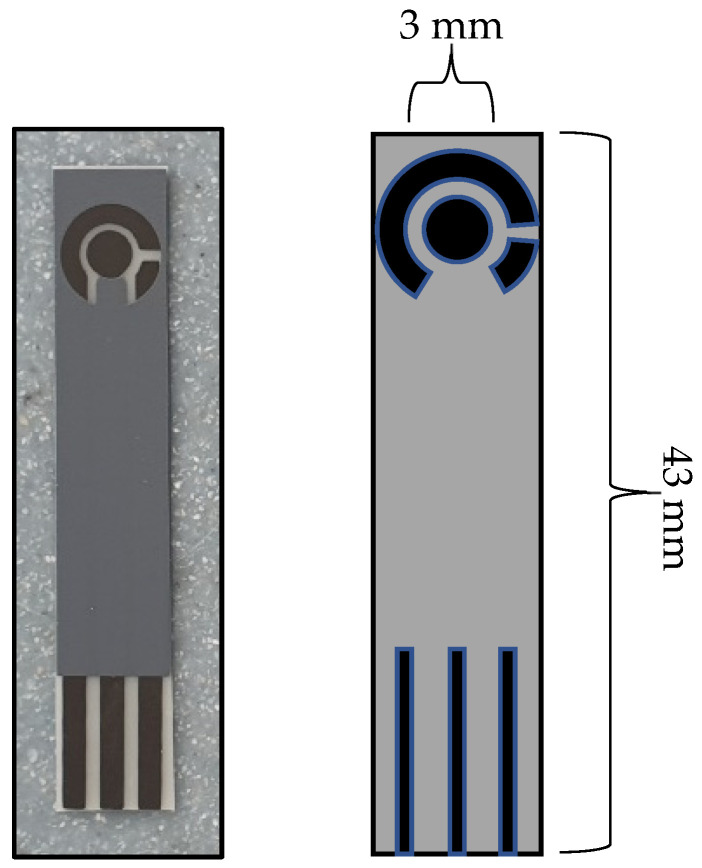
Schematic showing the complete C-MWCNT/DAO/EDC-NHS/GA SPEs (**Left**), and graphical depiction showing the sizes of the sensor (**Right**).

Figure 2 shows a schematic of the developed C-MWCNT/DAO/EDC-NHS/GA SPE that was used throughout this study.

**Figure 3:**
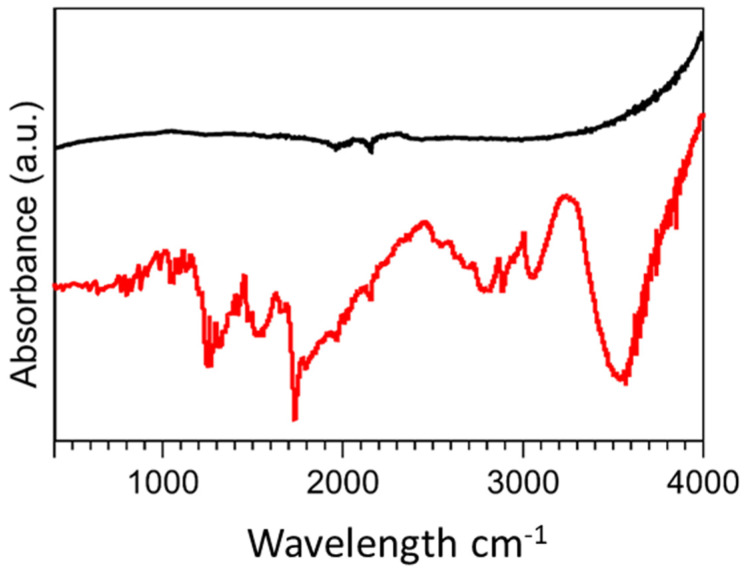
FTIR spectra of (Black) unmodified MWCNTs and (Red) diamine oxidase conjugated MWCNTs.

FTIR spectra were obtained in the range of 400 cm^−1^–4000 cm^−1^ to study the formation of new bonds on the MWCNT surface via the introduction of diamine oxidase and its crosslinkers (Figure 3). The unmodified MWCNTs demonstrated typical characteristic bonds respective of the photon modes of carbon nanomaterials at 1600 cm^−1^. For the functionalised MWCNTs, the organic bonds formed through the introduction of diamine oxidase and its associated conjugation biomolecules were demonstrated. At 3500 cm^−1^, weak C=O bonds were measured indicating the presence of carbonyl groups. Further functional groups at 2800 cm^−1^, 2363 cm^−1^, and 1100 cm^−1^ demonstrated C-H stretching, C=NH^+^, and C-N moieties, respectively, which were indicative of the ionic amine groups of the enzyme, diamine oxidase. Functionalized MWCNTs demonstrated peaks at 1715 cm^−1^ and 1300 cm^−1^, indicative of O-H stretching and C-O bonds, which were characteristic of COOH^−^ groups present in carboxyl functional groups. C=O bonds were identified between 1750 cm^−1^ and 1550 cm^−1^ which can be assigned to the carboxylic acid environment.

**Figure 4:**
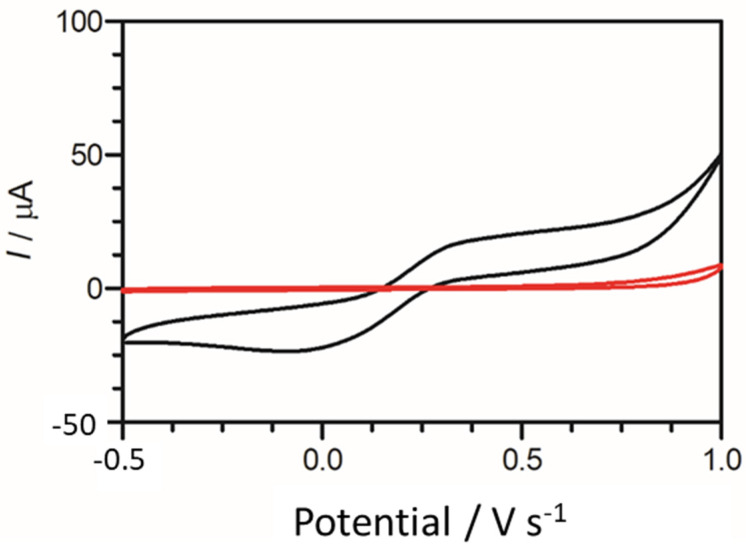
Cyclic voltammogram obtained using an unmodified carbon SPE (red scan) in the presence of 30 µg/mL of cadaverine solution in Britton–Robinson buffer at a scan rate of 100 mV s^−1^. Potential window −0.5 to 1.0 V s^−1^ vs. Ag/AgCl reference electrode. Cyclic voltammogram of cadaverine recorded utilising a C-MWCNT/DAO/EDC-NHS/GA working electrode vs. Ag/AgCl reference electrode (black scan) at a scan rate of 100 mV s^−1^.

To provide an initial baseline for cadaverine detection, an unmodified carbon graphitic ink SPE was used. It has been previously reported that cadaverine demonstrates a pair of redox peaks at concentrations ranging from 19.6 µM to 100 mM when measured using enzyme bio-transducers [32,44]. However, following the electrochemical analysis carried out in this work, cadaverine demonstrated no redox peaks at a concentration of 30 µg/mL when using an unmodified SPE vs. Ag/AgCl reference electrode (Figure 4). This may have been due to cadaverine presenting as an electrochemically inert molecule without the presence of an active site of a conjugated enzyme. Thus, under these conditions, the cadaverine molecule would not undergo a redox reaction without the addition of a catalyst molecule. The voltammetric response of cadaverine at the modified C-MWCNT/DAO/EDC-NHS/GA SPE was then explored, ensuring the same conditions were held as when measuring with unmodified SPEs. In contrast to the unmodified electrode, the results demonstrated a pair of redox peaks which denoted the electrochemical oxidation and reduction of cadaverine and H_2_O_2_, respectively, at the working electrode of the C-MWCNT/DAO/EDC-NHS/GA SPE. The demonstration of the peaks may have occurred in this instance since the cadaverine would be able to bind to the active site of the conjugated DAO enzyme located at the working electrode interface. This would have enabled the transport of electrons from the enzyme active site to the surface of the working electrode via the aid of the MWCNT scaffolding, resulting in the electrochemical measurements recorded.

**Figure 5:**
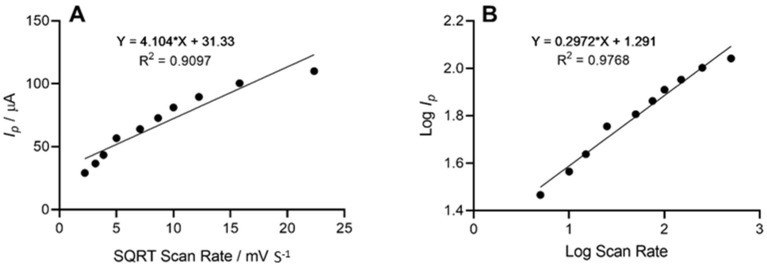
(**A**) Voltammetric anodic peak height of cadaverine expressed as a function of the square root of the scan rate. The line equation shows a linear relationship between peak height and square root scan rate with an R^2^ value of 0.9097. (**B**) Log peak current vs. log scan rate demonstrated a linear trend and a slope of 0.29 representing a diffusional controlled electrochemical process.

The height of the oxidation peak, which corresponded to the electrochemical oxidation of cadaverine as a result of enzyme–substrate interaction at the site of the working electrode, was next explored as a function of scan rate, whereby the anodic peak height (*I_p_*) vs. the square root of scan rate was plotted. This demonstrated a linear trend with respect to the peak current height of cadaverine. Further analysis depicted in the form of log peak current vs. log scan rate (Figure 5) demonstrated a slope of 0.29, which was found to be within the theoretical expected value of 0.5 for a diffusional controlled process at the surface of the working electrode, indicating an electrode structure that was non-porous [45]. Similar work has been previously conducted whereby the electrochemical oxidation of cadaverine was achieved via the active redox centre of the enzyme monoamine oxidase, a less-specific polyamine-oxidising enzyme than the enzyme utilised in this study [32]. The enzyme DAO demonstrates a much stronger affinity towards cadaverine than other similar biomolecules, and potentially results in increased sensitivity of the sensor, which would be a significant advantage in more complex solutions such as human saliva.

**Figure 6:**
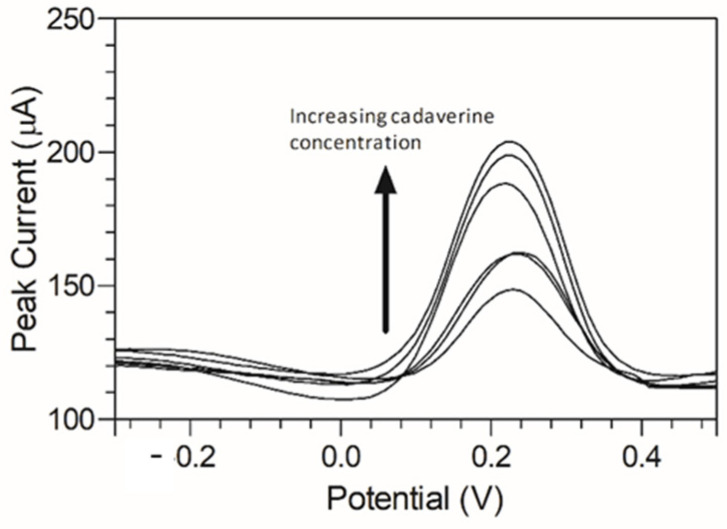
Anodic peak differential pulse voltammograms of cadaverine (3–150 µg/mL) using MWCNT/DAO/EDC-NHS/GA SPE with resulting peak current range of 32.22–43.13 µA vs. Ag/AgCl reference electrode in supporting Britton–Robinson buffer at pH 6.0.

Cadaverine has been previously demonstrated to have a potentially concentration-dependent influence on the disease state of periodontitis, ranging from mild to moderate and severe/advanced periodontitis. To emulate a periodontitis model of infection which centres around cadaverine, the concentrations of cadaverine chosen for detection corresponded to those in their respective periodontitis active disease stage [33]. Thus, electrochemical responses of the modified C-MWCNT/DAO/EDC-NHS/GA SPE platform were evaluated as a function of cadaverine concentration. Differential pulse voltammetry was chosen for this analysis due to its increased analytical peak sensitivity over other electroanalysis techniques. The single anodic peak of cadaverine in the response of the C-MWCNT/DAO/EDC-NHS/GA SPE was utilised and a DPV plot of cadaverine concentration vs. peak current was constructed (Figure 6).

**Figure 7:**
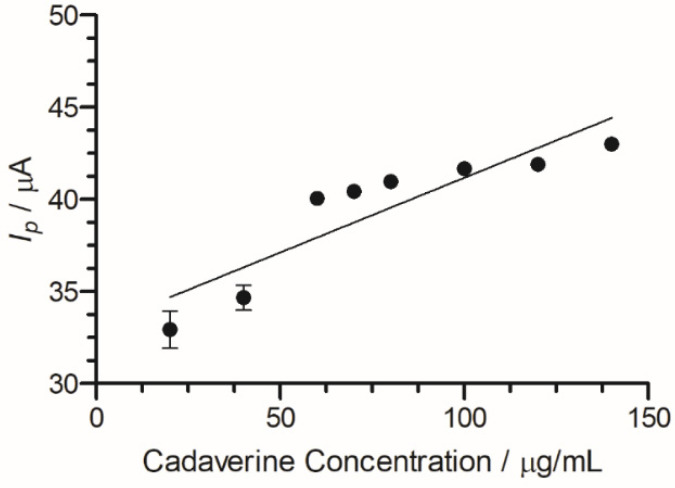
Cadaverine concentration (3–150 µg/mL) *I_p_* at C-MWCNT/DAO/EDC-NHS/GA SPE vs. Ag/AgCl reference electrode, with linear ranges identified at low and high concentrations of cadaverine.

A peak current range of 140–204 µA was determined at an increasing concentration of cadaverine (3–150 µg/mL). The concentration of cadaverine was plotted vs. peak current (Figure 7) and demonstrated a linear increase in analytical signal in response to increased cadaverine concentration. When the unmodified carbon SPE was used, little or no cadaverine was detected, similar to the cyclic voltammetric analysis demonstrated in Figure 4.

Using this information, the limit of detection for the C-MWCNT/DAO/EDC-NHS/GA SPE could be determined. The limit of the blank is defined as the highest concentration of apparent expected analyte concentration of replicates whereby no test analytes are to be found [46]. The mean blank value was obtained from the voltammetric response of a C-MWCNT/DAO/EDC-NHS/GA SPE in the absence of cadaverine. Changes to the current were recorded within the expected potential window (−0.3–0.5 V s^−1^) whereby the electrochemical oxidation and reduction of cadaverine would occur. Next, the limit of detection (LOD) was determined. The LOD is defined as the lowest concentration of analyte measured that is reliably distinguishable from the LOB. It is determined by calculating three times the standard deviation of the blank and for cadaverine this was 0.8 μg/mL [46]. The results demonstrated a LOD which was shown to be similar to previous works which measured cadaverine using a monoamine oxidase biosensor at 19.9 ± 0.9 µM [32]. Furthermore, the results showed increased sensitivity when compared to other works which demonstrated a detection limit of 50 µM [47]. Further studies demonstrated a linear range of 50 µM–1.6 mM for cadaverine and similar biogenic amines for a biosensor used on fish samples [48]. The increased sensitivity of this device may be hypothesised to be due to the modified enzyme/carbon nanotube surface of the working electrode allowing for an increased surface area to enable the enzyme–cadaverine interactions [39]. This resulted in the ability of the C-MWCNT/DAO/EDC-NHS/GA SPE to detect the electron transfer of the concentrations of cadaverine at lower levels than previously reported (Table 1).

**Figure 8:**
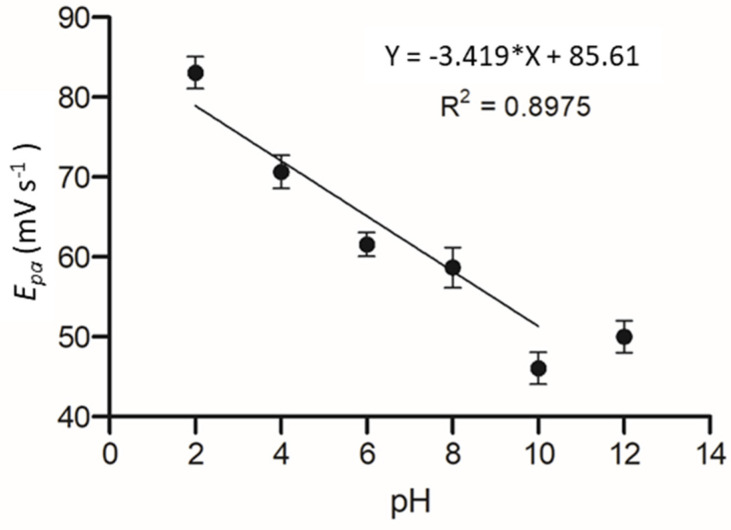
Plot of E_pa_ vs. pH at a range of 2 to 12 in Britton–Robinson buffered KCL solution.

The effect of pH on the electrochemical system was determined as it has been shown in previous works that pH can significantly alter the affinity of an enzyme to its substrate. The C-MWCNT/DAO/EDC-NHS/GA SPE was measured against a pH range of 2–12 and cyclic voltammetric profiles of cadaverine were assessed as a function of pH by plotting the oxidation peak of cadaverine vs. pH. It should be noted that the pK_a_ of cadaverine is 10.25 at 25 °C (Figure 8). A linear correlation was demonstrated between the increasing pH and the peak potential of cadaverine. The linearity of the system past the pK_a_ of cadaverine ceased, causing a shift in *E*_p_ towards higher values. The performance of the enzyme was strongly dependent on pH of the buffer solution, showing an increase in peak potential with an increase in pH.

**Figure 9:**
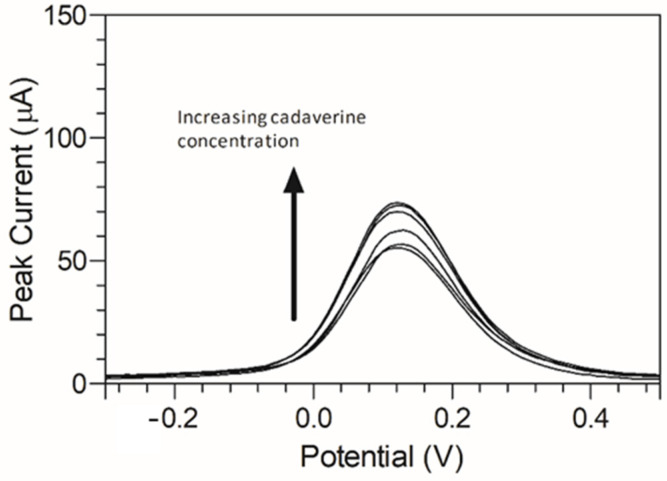
DPV response of cadaverine (30 µg/mL) at C-MWCNT/DAO/EDC-NHS/GA SPE in artificial saliva at a pH of 7.2 vs. Ag/AgCl reference electrode.

To better emulate the environment of the oral cavity, artificial saliva was used to evaluate the C-MWCNT/DAO/EDC-NHS/GA biosensor. The constituents of the saliva were previously elucidated [49]. The peak response of the cadaverine using the C-MWCNT/DAO/EDC-NHS/GA biosensor within artificial saliva was determined using DPV (Figure 9). When using the saliva, a narrower potential range was observed with a similar peak current response over multiple repeat measurements (Figure 9). This suggested that the biosensor would not be inhibited by interfering molecules present in human saliva. The biosensor demonstrated good efficacy when used in a simulated real-world environment, as the saliva of individuals may be used to potentially detect the level of cadaverine in the body as a rapid, non-invasive means of potential disease identification.

This work demonstrated the development of an MWCNT and DAO functionalised biosensor as a potential inexpensive and rapid alternative method of cadaverine detection. In addition, using this method precludes the requirement for sample pre-processing, a major advantage over current polyamine detection methods. This biosensor utilised DAO, a polyamine-specific enzyme, which was covalently crosslinked to MWCNTs and selectively detects cadaverine due to enzyme substrate specificity.

This method of detection can be compatible with a range of biomolecules by simply changing the detection enzyme in the biosensor system. The simplicity of fabrication and application lends itself to great interest within the healthcare environment due to low operational potential, low costs, and the benefit of real-time analysis, making it an ideal alternate device to current detection strategies.

## 4. Conclusions

This study tested the efficacy of a C-MWCNT/DAO/EDC-NHS/GA biosensing platform that was successfully developed for use in an electrochemical detection system for cadaverine. The incorporation of DAO onto C-MWCNTs demonstrated a viable method to measure the concentrations of cadaverine in both stock solutions and artificial saliva. Using electro-analytical techniques such as cyclic voltammetry and DPV, the C-MWCNT/DAO/EDC-NHS/GA biosensor demonstrated the ability to measure concentrations of cadaverine to as low as 0.8 μg/mL, which was measured in real time and showed limits of detection equivalent to, or lower than, that of current devices. Furthermore, the device was shown to respond to small changes in cadaverine concentrations, which may potentially be indicative of periodontal disease state changes within afflicted individuals. Thus, such a biosensor, which is cheap, readily producible, and produces measurements in real time, for use in cadaverine measurement has the potential to be developed for use in devices for the early detection of disease.

## Figures and Tables

**Table 1:** Previous reports of cadaverine, and other polyamine-based biosensors and their detection limits.

Sensor Detection Molecule	Detection Limit	Reference
Cadaverine	3 µg/Kg	[33]
Cadaverine and Putrescene	9.9 µM and 19.9 µM	[32]

## Data Availability

The data presented in this study are available on request from the corresponding author.

## References

[1] Sakanaka A., Kuboniwa M., Hashino E., Bamba T., Fukusaki E., Amano A. (2017). Distinct signatures of dental plaque metabolic byproducts dictated by periodontal inflammatory status. Sci. Rep..

[2] Rio B., Redruello B., Linares D.M., Ladero V., Ruas-Madiedo P., Fernandez M., Martin M.C., Alvarez M.A. (2019). The biogenic amines putrescine and cadaverine show in vitro cytotoxicity at concentrations that can be found in foods. Sci. Rep..

[3] Pugin B., Barcik W., Westermann P., Heider A., Wawrzyniak W., Hellings P., Akdis C.A., O’Mahony L. (2017). A wide diversity of bacteria from the human gut produces and degrades biogenic amines. Microb. Ecol. Health Dis..

[4] Babbar N., Murray-Stewart T., Casero R.A. (2007). Inflammation and polyamine catabolism: The good, the bad and the ugly. Biochem. Soc. Trans..

[5] Tomar P.C., Lakra N., Mishra S.N. (2013). Cadaverine: A lysine catabolite involved in plant growth and development. Plant Signal. Behav..

[6] Songa E., Okonkwo O. (2016). Recent approaches to improving selectivity and sensitivity of enzyme-based biosensors for organophosphorus pesticides: A review. Talanta.

[7] Stoner P. (1985). An improved spectrophotometric assay for histamine and diamine oxidase (DAO) activity. Agents Actions.

[8] Biji K.B., Ravishankar C.N., Venkateswarlu R., Mohan C.O., Gopal T.K. (2016). Biogenic amines in seafood: A review. J. Food Sci. Technol..

[9] Nalazek-Rudnicka K., Wasik A. (2017). Development and validation of an LC-MS/MS method for the determination of biogenic amines in wines and beers. Monatsh. Chem..

[10] Piermarini S., Volpe G., Federico R., Moscone D., Palleschi G. (2010). Detection of biogenic amines in human saliva using a screen-printed biosensor. Anal. Lett..

[11] Cho I.H., Kim D.H., Park S. (2020). Electrochemical biosensors: Perspective on functional nanomaterials for on-site analysis. Biomater. Res..

[12] Bhalla N., Jolly P., Formisano N., Estrela P. (2016). Introduction to biosensors. Essays Biochem..

[13] Yoo E.H., Lee S.Y. (2010). Glucose biosensors: An overview of use in clinical practice. Sensors.

[14] Rocchitta G., Spanu A., Babudieri S., Latte G., Madeddu G., Galleri G., Nuvoli S., Bagella P. (2016). Enzyme biosensors for biomedical applications: Strategies for safeguarding analytical performances in biological fluids. Sensors.

[15] Thangamuthu M., Gabriel W.E., Santschi C., Martin O.J.F. (2018). Electrochemical sensor for bilirubin detection using screen printed electrodes functionalized with carbon nanotubes and graphene. Sensors.

[16] Rowley-Neale S.J., Smith G.C., Banks C.E. (2017). Mass-producible 2d-mos2-impregnated screen-printed electrodes that demonstrate efficient electrocatalysis toward the oxygen reduction reaction. ACS Appl. Matr. Interfaces.

[17] Ferrari A.G.-M., Foster C.W., Kelly P.J., Brownson D.A.C., Banks C.E. (2018). Determination of the electrochemical area of screen-printed electrochemical sensing platforms. Biosensors.

[18] Hernández-Ibáñez N., García-Cruz L., Montiel V., Foster C.W., Banks C.E., Iniesta J. (2016). Electrochemical lactate biosensor based upon chitosan/carbon nanotubes modified screen-printed graphite electrodes for the determination of lactate in embryonic cell cultures. Biosens. Bioelectron..

[19] Kim B.C., Lee I., Kwon S.-J., Wee Y., Kwon K.Y., Jeon C., An H., Jung H.-T. (2017). Fabrication of enzyme-based coatings on intact multi-walled carbon nanotubes as highly effective electrodes in biofuel cells. Sci. Rep..

[20] Verma M.L., Naebe M., Barrow C.J., Puri M. (2013). Enzyme immobilisation on amino-functionalised multi-walled carbon nanotubes: Structural and biocatalytic characterization. PLoS ONE.

[21] Tîlmaciu C.M., Morris M. (2015). Carbon nanotube biosensors. Front. Chem..

[22] Yoo S., Baek Y.K., Shin S., Kim S., Jung H.T., Choi Y., Lee S.Y. (2016). Single walled carbon nanotube-based electrical biosensor for the label-free detection of pathogenic bacteria. J. Nanosci. Nanotechnol..

[23] Zhou Y., Fang Y., Ramasamy R. (2019). Non-covalent functionalization of carbon nanotubes for electrochemical biosensor development. Sensors.

[24] Vashist S.K., Zheng D., Al-Rubeaan K., Luong J., Sheu F. (2011). Advances in carbon nanotube based electrochemical sensors for bioanalytical applications. Biotechnol. Adv..

[25] Neupane S., Patnode K., Li H., Baryeh K., Liu G., Hu J., Chen B., Pan Y. (2019). Enhancing enzyme immobilization on carbon nanotubes via metal-organic frameworks for large-substrate biocatalysis. ACS Appl. Mater. Interfaces.

[26] Rochette J., Sacher E., Meunier M., Luong J. (2005). A mediatorless biosensor for putrescine using multiwalled carbon nanotubes. Anal. Biochem..

[27] Nguyen H., Lee S.H., Lee U.J., Fermin C.D., Kim M. (2019). Immobilized enzymes in biosensor applications. Materials.

[28] Putzbach W., Ronkainen N.J. (2013). Immobilization techniques in the fabrication of nanomaterial-based electrochemical biosensors: A review. Sensors.

[29] Zhu Z. (2017). An overview of carbon nanotubes and graphene for biosensing applications. Nanomicro Lett..

[30] Yates N., Fascione M.A. (2018). Parkin, Methodologies for “wiring” redox proteins/enzymes to electrode surfaces. Chemistry.

[31] Alexi N., Hvam J., Lund B., Nsubuga L., Oliveira Hansen R., Thamsborg K., Lofink F., Byrne D., Leisner J. (2021). Potential of novel cadaverine biosensor technology to predict shelf life of chilled yellowfin tuna (*Thunnus albacares*). Food Control.

[32] Henao-Escobar W., Domínguez-Renedo O., Asunción Alonso-Lomillo M., Julia Arcos-Martínez M. (2013). Simultaneous determination of cadaverine and putrescine using a disposable monoamine oxidase based biosensor. Talanta.

[33] Andörfer L., Holtfreter B., Weiss S., Matthes R., Pitchika V., Schmidt C.O., Samietz S., Kastenmüller G., Nauck M., Völker U. (2021). Salivary metabolites associated with a 5-year tooth loss identified in a population-based setting. BMC Med..

[34] García-Miranda Ferrari A., Rowley-Neale S.J., Banks C.E. (2021). Screen-printed electrodes: Transitioning the laboratory in-to-the field. Talanta Open.

[35] Kuboniwa M., Sakanaka A., Hashino S.E., Bamba T., Fukusaki E., Amano A. (2016). Prediction of periodontal inflammation via metabolic profiling of Saliva. J. Dent. Res..

[36] Ramenzoni L., Lehner M.P., Kaufmann M.E., Wiedemeier D., Attin T., Schmidlin P. (2021). Oral diagnostic methods for the detection of periodontal disease. Diagnostics.

[37] Campaña A.L., Florez S.L., Noguera M.J., Fuentes O.P., Ruiz Puentes P., Cruz P., Osma J.F. (2019). Enzyme-based electrochemical biosensors for microfluidic platforms to detect pharmaceutical residues in wastewater. Biosensors.

[38] Vashist S.K. (2012). Comparison of 1-ethyl-3-(3-dimethylaminopropyl) carbodiimide based strategies to crosslink antibodies on amine-functionalized platforms for immunodiagnostic applications. Diagnostics.

[39] Amin M., Abdullah B.M., Rowley-Neale S.J., Wylie S.R., Slate A.J., Banks C.E., Whitehead K.A. (2022). Diamine oxidase-conjugated multiwalled carbon nanotubes to facilitate electrode surface homogeneity. Sensors.

[40] Wickramathilaka M.P., Tao B.Y. (2019). Characterization of covalent crosslinking strategies for synthesizing DNA-based bioconjugates. J. Biol. Eng..

[41] Staros J.V., Wright R., Swingle D.M. (1986). Enhancement by N-hydroxysulfosuccinimide of water-soluble carbodiimide-mediated coupling reactions. Anal. Biochem..

[42] Fischer M.J. (2010). Amine coupling through EDC/NHS: A practical approach. Methods Mol. Biol..

[43] Noll T., Noll G. (2011). Strategies for “wiring” redox-active proteins to electrodes and applications in biosensors, biofuel cells, and nanotechnology. Chem. Soc.Rev..

[44] Spehar-Délèze A.-M., Almadaghi S., O’Sullivan C.K. (2015). development of solid-state electrochemiluminescence (ecl) sensor based on ru(bpy)_3_^2+^-encapsulated silica nanoparticles for the detection of biogenic polyamines. Chemosensors.

[45] Figueiredo-Filho L., Brownson D., Gómez-Mingot M., Iniesta J., Fatibello-Filho O., Banks C.E. (2013). Exploring the electrochemical performance of graphitic paste electrodes: Graphene vs. graphite. Analao.

[46] Armbruster D.A., Pry T. (2008). Limit of blank, limit of detection and limit of quantitation. Clin. Biochem. Rev..

[47] Male K.B., Bouvrette P., Luong J., Gibbs R.F. (1996). Amperometric biosensor for total histamine, putrescine and cadavarine using diamine oxidase. J. Food Sci..

[48] Vanegas D.C., Patiño L., Mendez C. (2018). Laser scribed graphene biosensor for detection of biogenic amines in food samples using locally sourced materials. Biosensors.

[49] Pytko-Polonczyk J., Jakubik A., Przeklasa-Bierowiec A., Muszynska B. (2017). Artificial saliva and its use in biological experiments. J. Physiol. Pharmacol..

